# Similarities and differences in placental development between humans and cynomolgus monkeys

**DOI:** 10.1002/rmb2.12522

**Published:** 2023-06-26

**Authors:** Shoma Matsumoto, Eiichi Okamura, Masanaga Muto, Masatsugu Ema

**Affiliations:** ^1^ Department of Stem Cells and Human Disease Models, Research Center for Animal Life Science Shiga University of Medical Science Otsu Japan; ^2^ Institute for the Advanced Study of Human Biology (WPI‐ASHBi) Kyoto University Kyoto Japan

**Keywords:** *Macaca fascicularis*, placenta, primates, stem cells, trophoblasts

## Abstract

**Background:**

The placenta is an extraembryonic organ, which is essential to maintain a normal pregnancy. However, placental development in humans is poorly understood because of technical and ethical reasons.

**Methods:**

We analyzed the anatomical localization of each trophoblastic subtype in the cynomolgus monkey placenta by immunohistochemistry in the early second trimester. Histological differences among the mouse, cynomolgus monkey, and human placenta were compared. The PubMed database was used to search for studies on placentation in rodents and primates.

**Main findings:**

The anatomical structures and subtypes of the placenta in cynomolgus monkeys are highly similar to those in humans, with the exception of fewer interstitial extravillous trophoblasts in cynomolgus monkeys.

**Conclusion:**

The cynomolgus monkey appears to be a good animal model to investigate human placentation.

## 1 INTRODUCTION

The placenta is an extraembryonic organ, which is essential to maintain a normal pregnancy in humans. The placenta forms a maternal–fetal junction and has several essential physiological functions, such as facilitating the supply of nutrition to the fetus, exchange of gases, and removal of wastes from the fetus.^1^ Therefore, placental dysfunction is directly associated with complications of pregnancy and fetal development in humans. The failure of trophoblast differentiation leads to serious diseases (e.g., intrauterine growth restriction and preeclampsia) in the fetus and mother.^2^, ^3^ However, the difficulty in obtaining placental samples in early normal and abnormal pregnancy at any gestation and the ethical restrictions for invasive study hinder the understanding of human placentation.

Eutherian mammals share similar fetal organ structures, but they show substantial diversity in the gross appearance of the placenta as follows. The representative classification includes discoid (rodents and primates), diffuse (horses and pigs), multicotyledonary (ruminants), and zonary (carnivores) (reviewed in Furukawa et al.).^4^ Among them, the mouse, which belongs to the discoid type (similar to humans), is widely used as a model for human placentation because of its ease of handling, developments in genome engineering techniques, and the presence of in vitro stem cell models.^5^ Previous studies using mouse models have shown mechanistic insights into early embryonic development and placental formation at the cellular and molecular levels during the past several decades. Recently, a large‐scale knockout analysis showed that early embryonic lethality accompanied by abnormal brain, heart, and vascular development was highly associated with placental dysfunction.^6^ However, there is a still evolutional distance between mice and the humans, indicating a need for animal models that are closer to humans.

In this review, we examine the utility of the cynomolgus monkey, which is a nonhuman primate (NHP), as an animal model for human placentation. Comparative studies among mice, cynomolgus monkeys, and humans have shown that the anatomical structures and trophoblast subtypes of the placenta in cynomolgus monkeys are highly similar to those in humans, with the exception of fewer interstitial extravillous trophoblasts in cynomolgus monkeys. The study of the cynomolgus monkey placenta with shallower extravillous trophoblast (EVT) invasion than that in humans may lead to the understanding of the molecular mechanism of placentation, by focusing on this difference in placental structure between the two species.

## 2 HUMAN PLACENTA

Human placental research has been conducted using clinical samples, including the term placenta and aborted conceptus. In vitro models, such as primary trophoblasts or choriocarcinoma cell lines, were the only option to investigate mechanistic insight into human placentation for many years because of the lack of adequate placental models. Nevertheless, many findings have been made in the human placenta.

### 2.1 Early placental formation

**FIGURE 1:**
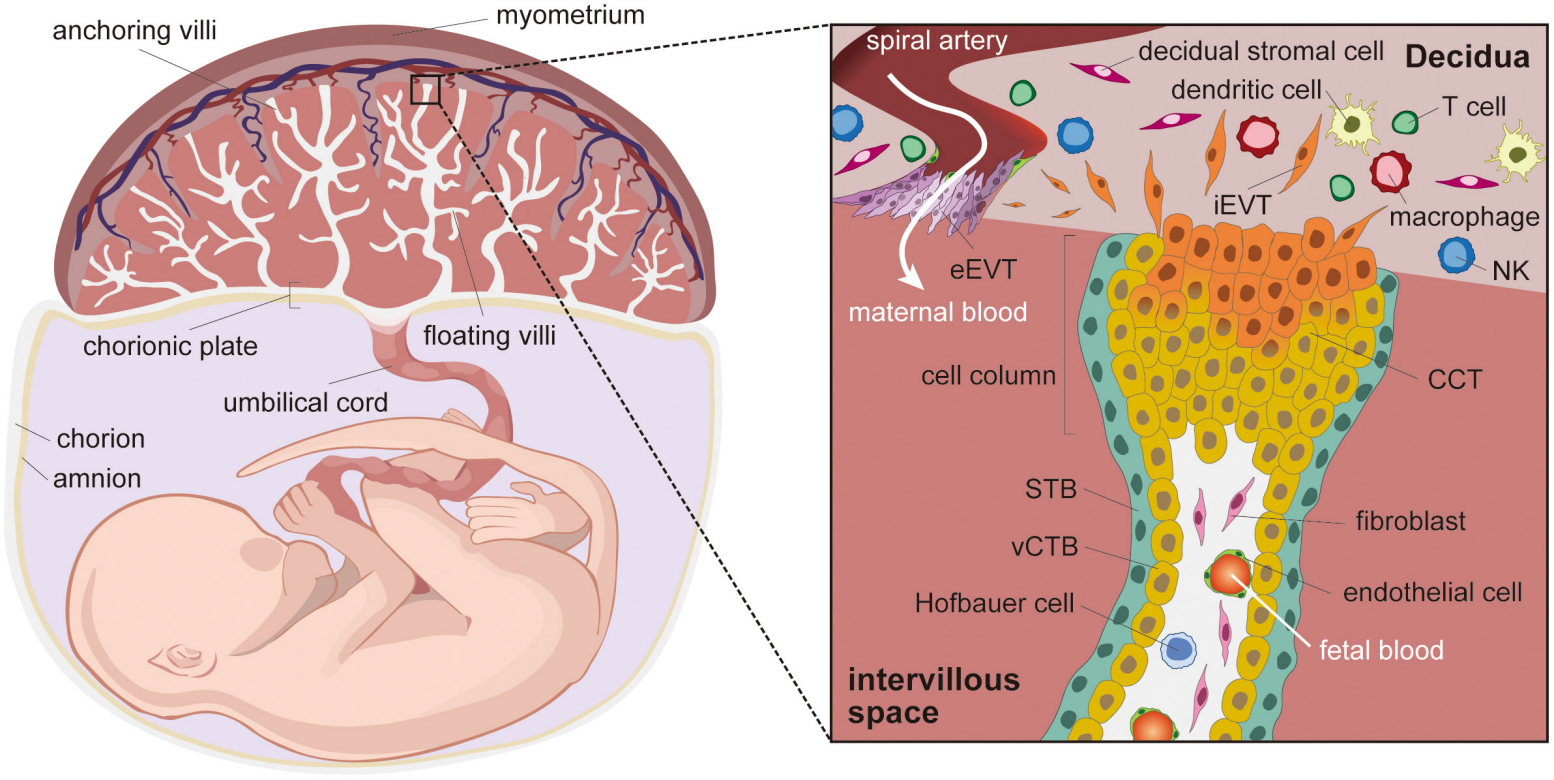
Structure of the primate placenta. Human and nonhuman primates form the discoid type of placenta, which is mainly composed of three trophoblastic subtypes, CTB (yellow), STB (green), and EVT (orange and purple). Proliferative CTBs are classified into two populations by their localization and gene expression pattern: vCTB and CCT. vCTBs and CCTs are considered as progenitors of STBs and EVTs, respectively. vCTBs and STBs generate a villous structure, and multinucleated STBs, which are formed by cell fusion, release pregnancy‐related hormones to maintain gestation. EVTs are differentiated from the tip of villi, and then they invade the maternal decidua to remodel spiral arteries. CCT, cell column trophoblast; CTB, cytotrophoblast; eEVT, endovascular extravillous trophoblast; EVT, extravillous trophoblast; iEVT, interstitial extravillous trophoblast; STB, syncytiotrophoblast; vCTB, villous cytotrophoblast.

**FIGURE 2:**
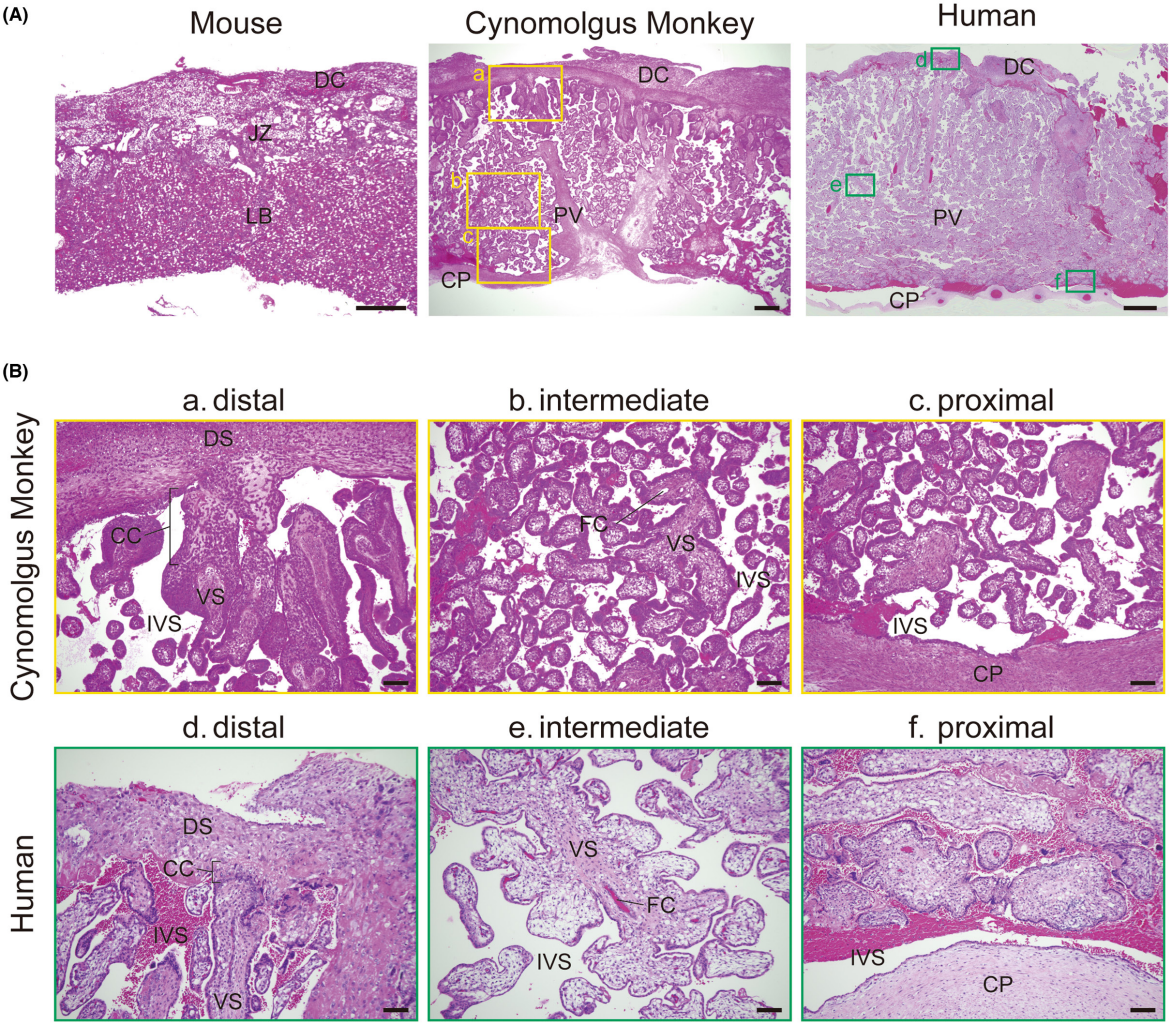
Histology of the cynomolgus monkey placenta. (A) Hematoxylin and eosin staining of mouse (E14.5), cynomolgus monkey (E61), and human (16 weeks) placentae. Scale bars of the mouse and cynomolgus monkey = 500 μm, human = 2 mm. (B) High magnification of the cynomolgus monkey and human placenta (related to panel A). Scale bar = 100 μm. CC, cell column; CP, chorionic plate; DC, decidua; FC, fetal capillary; IVS, intravillous space; JZ, junctional zone; LB, labyrinth layer; PV, placental villi; VS, villous stroma.

After fertilization, human oocytes develop into blastocysts composed of two cell lineages called the inner cell mass and trophectoderm (TE), which are considered to form the fetus and placenta, respectively (Carnegie stage 4).^7^, ^8^ Hatched blastocysts attach to the uterine wall from the polar side of the TE and invade the endometrium.^9^, ^10^, ^11^, ^12^ The TE immediately differentiates into the mononuclear trophoblast and primary syncytiotrophoblast (STB) at the postimplantation stage to facilitate the invasion of the embryo and to form the early placenta.^9^, ^10^, ^11^, ^12^, ^13^ The placenta is mainly composed of three trophoblast subtypes, which are the proliferative cytotrophoblast (CTB), multinucleated STB, and invasive EVT.^10^, ^11^, ^12^, ^13^, ^14^ Moreover, the CTB is classified into the villous cytotrophoblast (vCTB) and cell column trophoblast (CCT) by their anatomical localization and gene expression profile (Figure 1).^10^, ^12^, ^14^ The inner layer of vCTBs and outer layer of STBs form villi with stromal cells inside, and the villi elongate to the maternal side. The primary placenta with a villous structure forms at approximately fourth week after fertilization. Cell columns appear at the tip of the villi, and are called anchoring villi, which attach to the maternal decidua. Invasive EVTs, which are differentiated from CCTs, migrate toward the maternal side to remodel the maternal spiral artery (SpA) (Figures 1 and 2A,B). Maternal blood is supplied into the intravillous space at approximately the 10–12 weeks of gestation.^15^

### 2.2 Features of trophoblastic subtypes

Proliferative vCTBs differentiate toward multinucleated STBs, which cover the entire surface of placental villi through cell fusion.^16^ At the first step of trophoblastic cell fusion, adenylate cyclase increases intracellular cAMP to activate protein kinase A, which phosphorylates several transcription factors, such as *GCM1*.^16^ *GCM1* enhances human endogenous retrovirus envelope gene expression of *ERVW‐1* and *ERVFRD‐1*, which are known as Syncytin‐1 and ‐2, leading to cell fusion.^17^, ^18^, ^19^ STBs contributes to maintain normal gestation by releasing pregnancy‐specific hormones, such as chorionic gonadotropin (CG), placental lactogen, and steroid hormones.^20^ These hormones contribute to normal pregnancy through the promotion of STB formation in an autocrine–paracrine manner.^16^, ^20^ In fact, primary CTBs isolated from the human placenta easily differentiate to STBs by CG treatment.^21^

Intervillous oxygen tension rises from 2% to 3% oxygen at 8–10 weeks of gestation to more than 6% oxygen after 12 weeks.^22^, ^23^ While primary CTBs differentiate toward the STBs under normoxia and/or cAMP treatment,^24^, ^25^ isolated CTBs show EVT‐like features with *HIF1α* upregulation under hypoxia.^26^ Human placental villi explant culture also shows that EVT differentiation is enhanced by low oxygen tension.^27^ During EVT differentiation, CTBs at the anchoring cell column change their integrin molecule pattern from proximal to distal CCTs (from the fetal to the maternal side). vCTBs strongly express the integrin α6 (ITGA6)/B4 complex, but the expression level gradually decreases at the distal part. In contrast, the ITGA5/B1 and ITGA1/B1 complexes are upregulated at the distal cell column and decidual zone.^28^ Although invasiveness of EVTs is acquired during epithelial‐to‐mesenchymal transition,^29^ interstitial EVTs (iEVTs) invade the uterine SpA with the removal of vascular smooth muscle cells and endothelial cells. Endothelial cells are then replaced with endovascular EVTs (eEVT).^3^, ^30^, ^31^ SpA remodeling leads to maternal blood flow in the intervillous space from the end of the first trimester to the mid‐second trimester to supply nutrition and increase oxygen tension.

Not only oxygen tension, but also maternal cell‐derived soluble factors, function in the maintenance and controlling the differentiation of trophoblasts. *WNT5A* and *IL33* secreted from decidual macrophages enhance vCTB and CCT proliferation in first trimester villous explant culture.^32^, ^33^ *EGF* and *HB‐EGF*, which are expressed by decidual fibroblasts, are also associated with an increase in CCT proliferation.^34^ Moreover, self‐renewal of CTBs are regulated by Hippo, Notch, and Wnt pathways.^19^, ^35^ *TEAD4* and *TP63* contribute to vCTB cell proliferation, and the NOTCH1 intracellular domain prevents EVT differentiation by maintaining CCT proliferation.^36^, ^37^ Canonical Wnt signaling plays a crucial role in not only EVT differentiation, but also the regulation of trophoblast motility.^35^ A study on a placental organoid model showed that activation and deactivation of Wnt signaling were essential for EVT differentiation.^38^ The failure of EVT differentiation causes placental dysfunction and leads to diseases in pregnancy, such as preeclampsia.^39^ Moreover, overexpression analyses have shown that *TP63* inhibits EVT migration and represses EVT marker genes, such as *ITGA1*, *ITGA5*, *ITGB1*, and *MMP2*.^40^ The mechanistic insights into human placentation described above are mostly based on in vitro experiments with primary cells, stem cells, and organoids. In vivo experiments that investigate placentation invasively or perturbed gene function are limited in humans because of ethical and technical reasons.

## 3 MOUSE PLACENTA

### 3.1 Anatomical insights

Mice are widely used as an animal model for understanding human placentation because mice show similar physiological features of the placenta to those in humans, and manipulating gene function is relatively easy in mice. Innovative genome engineering techniques and the existence of useful stem cell lines have provided considerable knowledges for early embryonic development and placentation. However, there are substantial differences in the developmental mechanism and anatomical features between mice and humans.^41^, ^42^ Blastocysts attach to the endometrium from the mural side in the mouse, but attach from the polar side in humans.^8^ The extraembryonic ectoderm and ectoplacental cone, which are characteristic structures in mice, but not present in humans, give rise to the placenta.^8^, ^42^, ^43^ In mice, the placenta is mainly composed of two layers called the labyrinth layer and junctional zone (Figure 2A). There are also differences in trophoblast subtypes regarding their features and marker genes between mice and humans. The murine placenta is composed of trophoblast giant cells, which have polyploid nuclei resulting from endoreduplication, glycogen trophoblasts, their progenitor spongiotrophoblasts, and STBs.^42^ The wide variety of trophoblastic subtypes cause difficulty in comparing mouse and human placentae because of the differences in gene expression profiles and their cellular functions.

### 3.2 Gene regulatory networks

Research on determining gene regulatory networks related to placentation using early mouse embryos and trophoblast cell lines has been conducted.^44^ Even though there might be species differences at the cellular level, knowledge from mouse experiments help understanding of placental formation in humans. Recent studies have shown that the deactivation of the Hippo signaling pathway is commonly observed in mouse, rat, cow, and human TE.^45^, ^46^ Mouse and human trophoblasts share similar epigenetic features. Specifically, tissue‐dependent and differentially methylated regions between trophoblast and embryonic lineages are conserved between them.^47^ In addition, trophoblast cell‐specific large heterochromatin architecture with a high degree of histone H3.1/3.2 and H3K9me3 accumulation (THDs) are found in both species.^48^

However, there are clear differences related to gene regulation networks between mice and humans as follows. First, *Cdx2* is progressively restricted to the outer cells in the morula.^49^ In humans, *CDX2* expression is detectable only after the early blastocyst stage.^50^ Moreover, while *Cdx2* functions as a master regulator by enhancing a set of key trophoblast genes, such as *Eomes*, *Tfap2c*, *Gata3*, *Elf5*, and *Ets2*, to maintain the proliferation and inhibition of differentiation of trophoblast stem cells (TSCs),^42^, ^44^ *CDX2* is not expressed in human TSCs (hTSCs).^51^ Second, although a well‐known signaling pathway, Activin/Nodal, plays a crucial role in maintaining stemness of TSCs in the mouse, Activin induces EVT differentiation from primary CTBs in humans.^52^ Finally, the imprinted X chromosomal inactivation mechanism observed in the mouse placenta is not fully preserved in humans.^53^

These different gene regulatory networks and signaling pathways might provide cues to solving the evolutionary differences between mice and humans. Taking into consideration that there are similarities and differences in epigenetic regulation between the mouse and human placenta, other animal models that are closer to humans are required to understand human placentation.

## 4 NONHUMAN PRIMATE MODEL

### 4.1 Cynomolgus monkeys as a human model

NHPs including New World monkeys (e.g., common marmosets) and Old World monkeys (e.g., rhesus monkeys and cynomolgus monkeys), which belong to macaques, are considered one of the most useful animal models to understand the mechanism of human embryogenesis and diseases. They are useful models because they show highly similar anatomical, physiological, and genomic features to those in humans. In particular, cynomolgus monkeys are considered a useful model because they can be bred throughout the year, and human disease models can be created by the lentivirus and CRISPR‐Cas9 systems.^54^, ^55^

### 4.2 Cynomolgus monkey placenta

While approximately 280 days are required for human gestation, the gestation time in cynomolgus monkeys is approximately 160 days.^56^ After implantation, the TE rapidly differentiates into primitive mononuclear CTBs and STBs, and trophoblast cells subsequently invade toward the uterus, similar to humans.^57^ In contrast to humans, the cynomolgus monkey embryo barely invades the maternal uterus; therefore, placental formation occurs on the surface of the endometrium.^58^ Although the primate placental structure is completed by the end of the first trimester, trophoblasts further invade the maternal side to remodel the SpA with the formation of placental villi.

**FIGURE 3:**
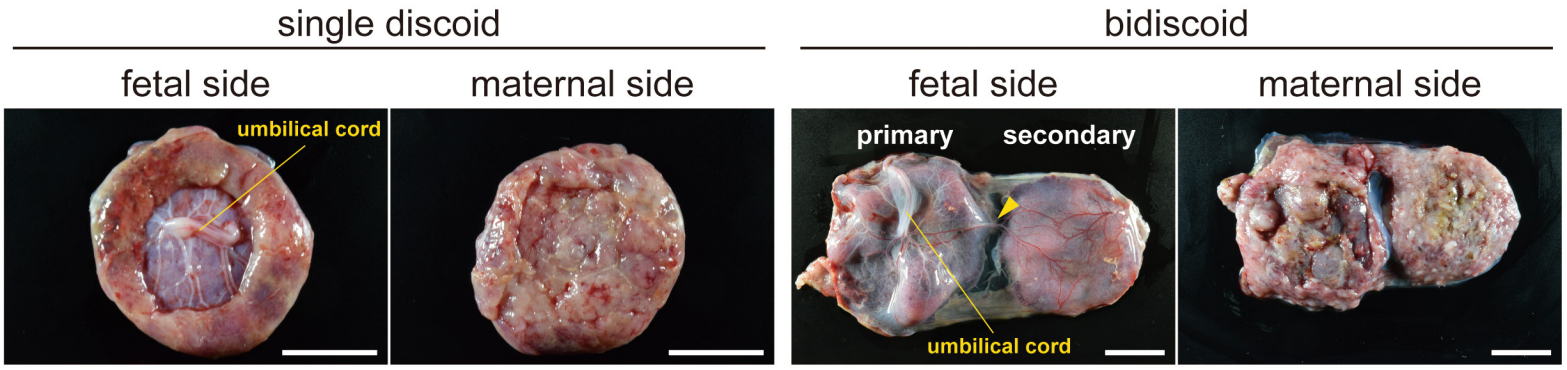
Images of single (E57) and bidiscoid (E58) cynomolgus monkey placentae. These placentae were obtained by cesarean section after multiple embryo transfer into a recipient monkey. The yellow arrowhead shows the vessel that provides blood flow into the secondary placenta. Scale bar = 2 cm.

Human and macaque placentae are the discoid type, but both single and bidiscoid placentae are observed in cynomolgus monkeys during their pregnancy (Figure 3). The secondary placenta is linked to a primary placenta via a fetal vessel, and there is no difference in morphological appearance between the primary and secondary placentae (Figure 3). Human and cynomolgus monkey placentae share a common tree‐like villous structure, which is derived from vCTBs and STBs. Major placental villi develop from the chorionic plate, and a number of small villi branch from them. When these villi expand from the fetal to the maternal side, another CTB subtype, CCTs, appear at the tip of villi (Figure 2B). CCTs reach the maternal decidua, and CCTs differentiate into EVTs to induce maternal blood flow into the placenta.

**FIGURE 4:**
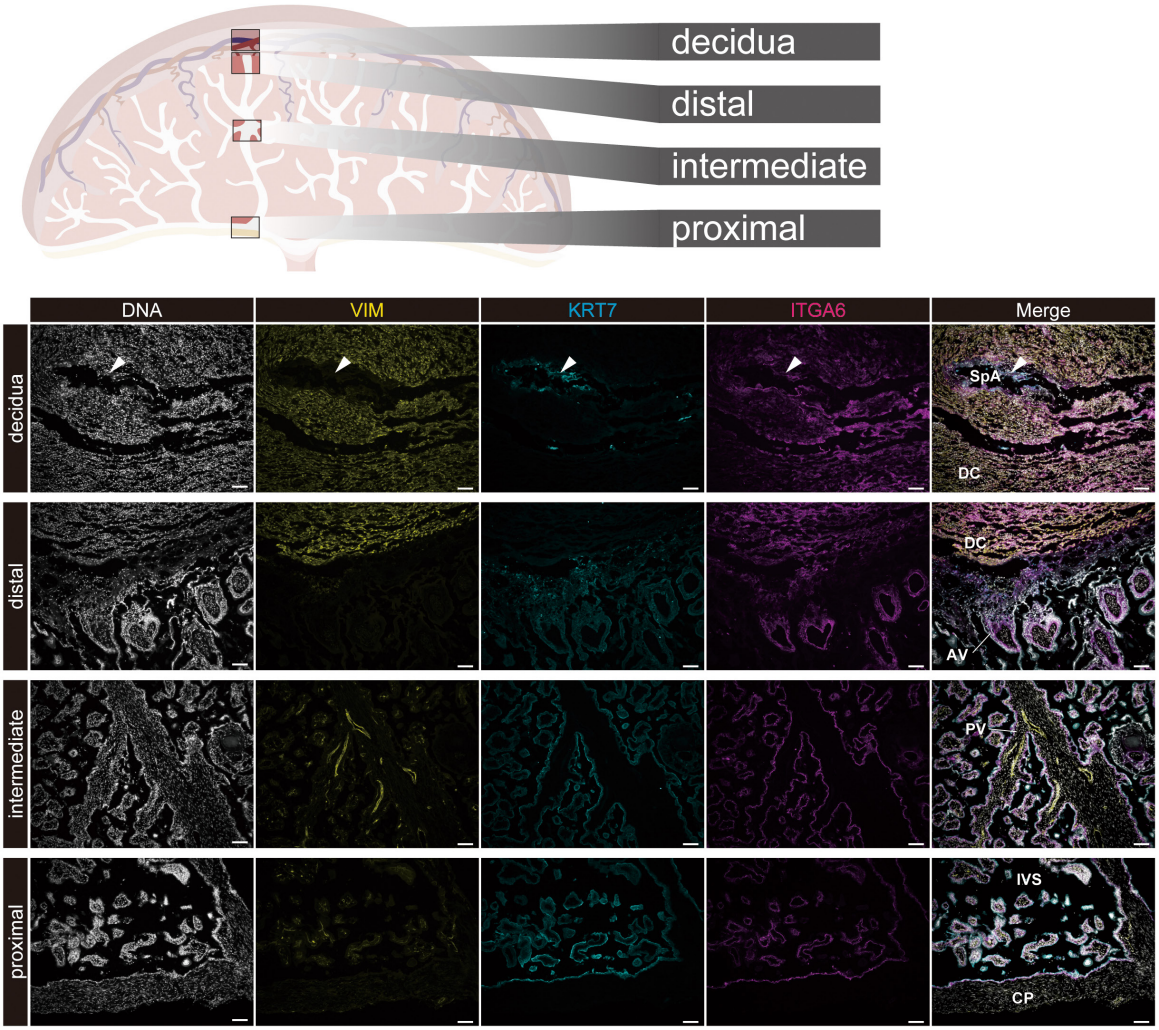
Immunohistochemistry for each part of the cynomolgus monkey placenta (E61). KRT7 is a pan‐trophoblast marker. VIM highlights stromal cells and endothelial cells. ITGA6 is expressed in CTBs and in some decidual cells. KRT7+/ITGA6+ cells are cytotrophoblasts. KRT7+ cells localized at the outer layer of placental villi are STBs. KRT7+ cells at the tip of anchoring villi are CCTs. White arrowheads show replaced extravillous trophoblasts at SpA. Scale bar = 100 μm. AV, anchoring villi; CP, chorionic plate; DC, decidua; IVS, intravillous space; PV, placental villi; SpA, spiral artery.

**FIGURE 5:**
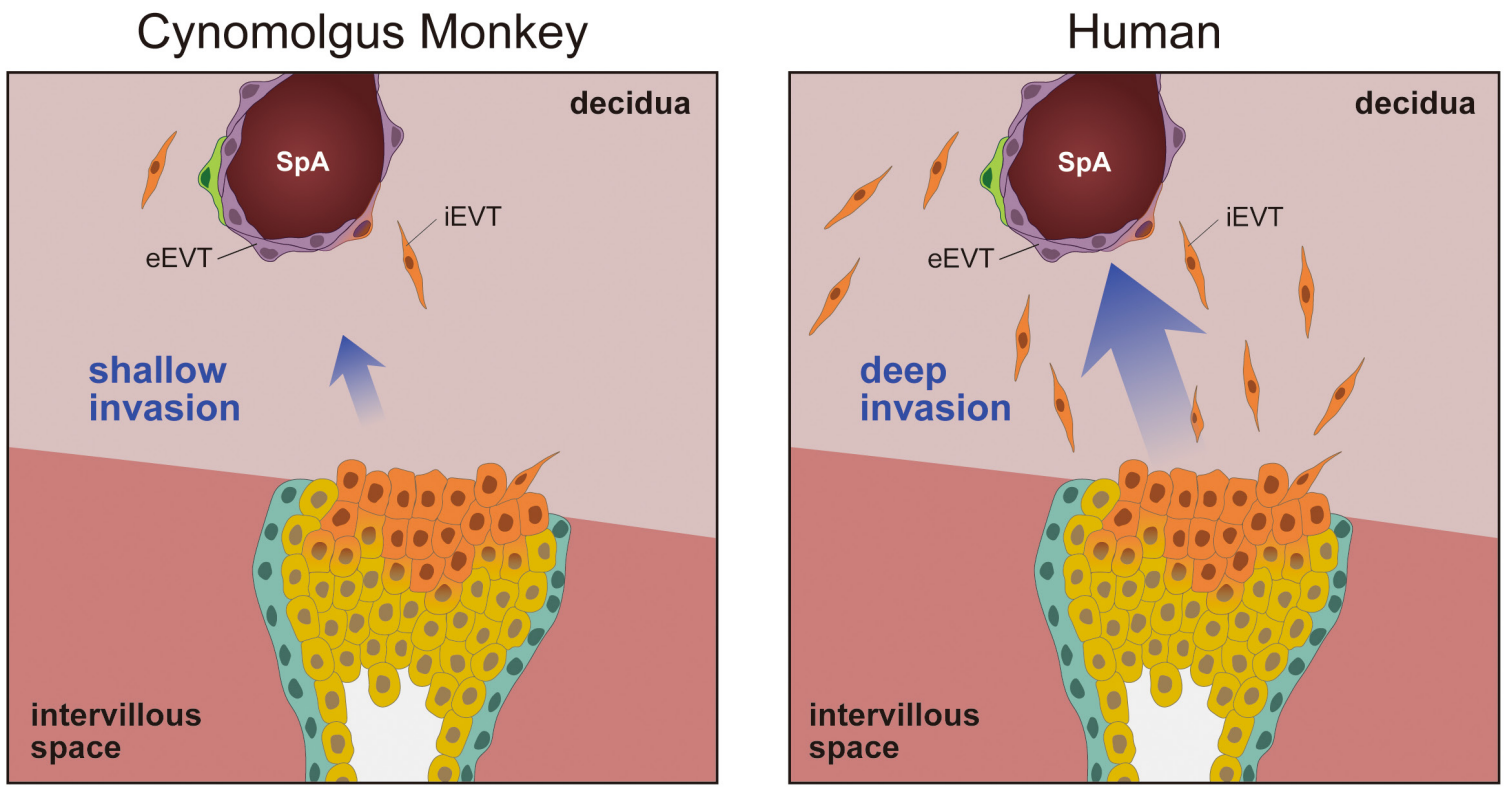
Invasiveness of cynomolgus monkey and human EVTs. Although a large number of iEVTs are observed at the decidua in humans, a few iEVTs are seen in cynomolgus monkeys. However, maternal endothelial cells at the spiral artery are replaced with eEVTs in cynomolgus monkeys and humans. eEVT, endovascular EVT; EVT, extravillous trophoblast; iEVT, interstitial EVT; SpA, spiral artery.

To reveal the precise placental structure in the cynomolgus monkey, we performed immunohistochemistry to highlight each trophoblastic subtype at the early second trimester. Immunohistochemistry showed that placental villi were elongated from the chorionic plate (Figure 4). Cynomolgus monkey placental villi were KRT7^+^/ITGA6^+^ in the inner layer of the vCTB and KRT7^+^/ITGA6^−^ in the outer layer of the STB, similar to in humans. VIM^+^ mesenchymal and endothelial cells were localized inside of the placental villi. In CTBs at anchoring villi around the distal part of the placenta, ITGA6 expression was gradually decreased from the fetal to maternal side, suggesting that integrin switching also occurs during EVT differentiation in the cynomolgus monkey. Although deep EVT invasion into the decidual stroma is shared with humans and great apes, Old World monkeys, including cynomolgus macaques, show much shallower trophoblast invasion.^59^, ^60^ Our results also indicated that few EVTs were localized in the decidual region, which suggests that cynomolgus monkey EVT shows shallower invasion than that in humans (Figures 4 and 5). A previous study showed that, in macaques, few trophoblast cells invade the maternal side compared with humans.^61^ Carter et al. also found the lack of an iEVT population in the rhesus monkey, which is a macaque.^57^ However, we found that endothelial cells at the SpA were replaced with KRT7^+^ endovascular EVTs in cynomolgus monkeys (Figure 4, arrowheads), which indicated sufficient maternal blood supply for fetal development. EVT differentiation and/or the regulation of EVT invasion in cynomolgus monkeys may be distinct from those in humans, and few iEVTs in the cynomolgus monkey placenta contribute to a lower number of total EVTs (Figure 5).

The placenta functions as the maternal–fetal junction. Therefore, the placenta has a unique immunotolerance system to protect against maternal immune cells. Human leucocyte antigen (HLA) is classified into class I, comprising *HLA‐A*, *‐B*, and *‐C*, and class II, comprising *HLA‐DP*, *‐DQ*, and *‐DR*. One of the HLA class I molecules, *HLA‐C*, is lacking in macaques.^62^ A placenta‐specific nonclassical HLA class I molecule, *HLA‐G*, is generally used as an EVT marker in humans.^63^ *Mamu‐AG* was identified as the ortholog of *HLA‐G* in the rhesus monkey because *Mamu‐G* is a pseudogene in Old World monkeys.^64^ Although Slukvin et al. succeeded in generating a Mamu‐AG‐specific monoclonal antibody, *Mamu‐AG* and *Mafa‐AG*, which is the ortholog of *Mamu‐AG* in cynomolgus monkeys, were also expressed in STBs at placental villi.^65^, ^66^ Therefore, the discovery of new EVT markers in cynomolgus monkeys is required.

## 5 IN VITRO MODEL

### 5.1 Mouse trophoblast stem cell lines

To investigate gene functions and molecular mechanisms for placental tissue, the in vitro culture system is used to understand biological progress. TSCs were first established under FGF4‐treated culture condition as an in vitro model of placental cells in mice.^67^ This useful tool revealed further molecular mechanisms and signaling pathways for trophoblast cell maintenance and differentiation in the mouse placenta.^42^, ^68^ Mouse TSCs (mTSCs) have the potential to differentiate all trophoblastic subtypes. However, interestingly, TSC lines can be established from not only the TE, which is the outer layer of the blastocyst, of preimplantation embryos, but also from extraembryonic ectoderm at embryonic day 6.5 (E6.5) and the chorion at E7.5–8.5 postimplantation embryos.^67^, ^69^ mTSCs were originally maintained in mouse embryonic fibroblast‐conditioned medium containing 20% serum. However, chemically defined culture methods, which improve the maintenance, derivation efficiency, and differentiation capacity of mTSCs, are now available.^70^, ^71^ Recently, one of the chemically defined culture conditions was further optimized, and then TE stem cells, which have similar features to polar TE, were established.^72^ These powerful in vitro tools are expected to help understand molecular functions for not only postimplantation events, but also for early embryogenesis.

### 5.2 Primate trophoblast stem cell lines

In contrast to rodents, establishing TSC lines in primates took a long time. Human primary mononuclear CTBs were successfully isolated from the term placenta using the Percoll gradient method, but trophoblasts could not be maintained because they immediately differentiated toward STBs.^24^ Even if the mTSC condition adapts to human trophoblast culture, it fails to keep an undifferentiated lineage.^73^ These results suggest that there are some differences in not only the appearance and structure of the placenta, but also in trophoblastic features, such as cytokine requirements, between rodents and primates. Several types of human cellular models have been developed to date. Choriocarcinoma cell lines, such as BeWo, JEG‐3, and JAR, which are transformed cell lines, and HTR‐8/SVneo and SGHPL‐4 have been widely used as human placental models for a long time. However, there are limitations of these models because they are derived from tumors or lack trophoblastic features.^42^ To induce trophoblastic lineage, transdifferentiation techniques, such as BMP4 treatment in human embryonic stem cells, have also been developed.^74^ These models also show a trophoblast‐like phenotype, but they cannot mimic primary trophoblastic features observed in vivo. To solve this issue, the following criteria to define early trophoblast cells in humans were reported: mononuclear trophoblasts at the first trimester should express adequate markers, such as *KRT7*, *TFAP2C*, and *GATA3*, and primate placenta‐specific nonprotein‐coding microRNAs that are located on chromosome 19.^75^ Epigenetic features, hypomethylation of the *ELF5* promoter region, and a lack of HLA class I molecule expression are also markers of first trimester mononuclear trophoblasts.^75^ Twenty years after the establishment of mTSCs, hTSCs, which fulfilled the criteria mentioned above, were established from blastocysts and isolated CTBs in the early placenta.^51^ Unlike the mTSC culture condition, hTSCs are maintained under low concentrations of serum and several small molecular compounds, such as TGFβ inhibitors, Wnt activator, HDAC inhibitor, and ROCK inhibitor.^51^ Taking into consideration the CTB‐like features with the potential to differentiate into STBs and EVTs, hTSCs are expected to be a more appropriate culture model for human placental development. Notably, hTSCs lose trophoblast‐specific H3K9me3 domains, namely THDs, during their establishment^48^ in contrast to mTSCs that maintain THDs. The lack of THDs in hTSCs might be caused by valproic acid, which is a histone deacetylase inhibitor that is widely used to erase epigenetic memory. In a recent report, hTSCs were established from human‐naïve pluripotent stem cells via a TE‐like state.^76^ These in vitro culture models are expected to help determine the molecular mechanism of early placentation in humans.

As an NHP placental model, trophoblast cells are isolated from rhesus monkey blastocysts. These cells can be maintained for more than 23 passages. *CG* and *OCT4*, which are STB and inner cell mass markers, respectively, are also expressed under the undifferentiated condition.^77^ Recently, cynomolgus monkey TSC lines, called macTSCs, were derived from blastocysts under the optimized mTSC culture condition.^78^ Although macTSC growth is enhanced by FGF4 similar to that in mTSCs, macTSCs fulfill the criteria reported by Lee et al. and have the potential to differentiate into the trophoblastic subtypes STB and EVT. Interestingly, macTSCs contribute to only the TE as shown by the xenogeneic chimera blastocyst formation assay.^78^ However, differentiation by cAMP treatment into STBs is not efficient. After the derivation of macTSCs, other macaque TSC lines were generated from rhesus monkey and cynomolgus monkey placental tissues using the hTSC culture condition.^79^ STB and EVT‐like differentiation is induced under the same conditions as those in hTSC differentiation, while multinucleated STB formation efficiency in the two‐dimensional culture condition and gene expression patterns in the stem cell state and EVTs are unlike those in hTSCs and macaque placental tissues. Optimizing TSC culture conditions in NHPs is essential to develop them as a useful in vitro model.

### 5.3 Organoids

Recently, a new three‐dimensional culture system called organoid culture has been developed using TSCs in humans. Placental organoids contain both CTB and STB populations, such as placental villi.^38^, ^80^, ^81^ Another trophoblastic subtype, EVT, is easily inducible from organoids by changing their culture condition, and the invasiveness of EVT organoids is enhanced by endometrial cells.^81^ Improvement of the method to mimic placental structure in vitro is required because the localization of each trophoblast subtype is unlike that in vivo.

Although placental organoids are expected to be a useful tool to examine postimplantation trophoblast features in vitro, a preimplantation model called the blastoid model has been newly generated. Blastoids, which have a blastocyst‐like structure and features, are generated by the aggregation of embryonic stem cells and TSCs.^82^ This structure can be induced only using naïve pluripotent stem cells in mice and humans.^83^, ^84^, ^85^, ^86^ This innovative tool will enhance the understanding of human embryogenesis, but in vivo experiments are necessary to identify the mechanism of early placentation at the postimplantation period.

## 6 CONCLUSION

Cynomolgus monkeys are a useful animal model to provide novel insight into embryonic and placental development in primates because of ethical and technical obstacles in humans. While these two species show high similarities, there are some differences in early development between cynomolgus monkeys and humans (e.g., the invasiveness of trophoblasts into the decidua). Therefore, identifying the mechanism underlying these differences may answer the fundamental question of what is involved in the inherent anatomical structure of the placenta in humans.

In this article, we summarize multispecies comparisons mainly in trophoblast cells in placental development. A recent single‐cell RNA sequencing analysis of the human placenta comprehensively showed the cell population, gene expression profile, and cell–cell interaction of all subtypes, highlighting the mechanism of immunotolerance that plays an important role in normal pregnancy.^87^, ^88^ Determining the complex mechanism of the interaction between trophoblast cells and immune cells in cynomolgus monkeys may lead to better understanding of human infertility.

## FUNDING INFORMATION

This work was supported by JSPS KAKENHI Grant Numbers 22K15028 (to S.M.), 23H03860 (to E.O.), 20K15699 (to M.M.), 20H05359, and 22H04669 (to M.E.), and the Naito Foundation (to M.E.). The funding source had no role in the design, practice, or analysis of this study.

## CONFLICT OF INTEREST STATEMENT

Authors declare no conflict of interests for this article.

## ETHICS STATEMENT

All animal experiments were conducted by following the guideline approved by the Institutional Animal Care and Use Committee of Shiga University of Medical Science (21‐002 and 22‐025).
